# Supplementary material for: Postoperative analgesia using dezocine alleviates depressive symptoms after colorectal cancer surgery: A randomized, controlled, double-blind trial
Source: PLoS One. 2020 May 26;15(5):e0233412. doi: 10.1371/journal.pone.0233412 (PMC7250456; doi:10.1371/journal.pone.0233412)
Supplement: Attachment — Submitted filename: Response to Reviewers.doc [file pone.0233412.s004.doc]

March 26, 2020

Emily Chenette
Staff Editor

*PLOS ONE*

Dear Editor:

We wish to re-submit the manuscript titled “Postoperative analgesia using dezocine alleviates depressive symptoms after colorectal cancer surgery: A randomized, controlled, double-blind trial.” The manuscript ID is PONE-D-19-20751.

We thank you and the reviewers for your thoughtful suggestions and insights. The manuscript has benefited from these insightful suggestions. I look forward to working with you and the reviewers to move this manuscript closer to publication in *PLOS ONE.*

The manuscript has been rechecked and the necessary changes have been made in accordance with the reviewers’ suggestions. All changes in the revised document are marked in red font. The responses to all comments have been prepared and are given below.

In line with the journal’s requirements and your recommendations, we deposit our laboratory protocols in protocols.io where a protocol can be assigned its own identifier (DOI). DOI link: <http://dx.doi.org/10.17504/protocols.io.bdysi7we> .

Thank you for your consideration. I look forward to hearing from you.

Sincerely,

Hong Li

****************************************************

**Reviewer #2:**

1.  Thank you for resubmitting the manuscript to PLOS ONE. I think you have read the reviewer's comments carefully and made modifications. As a whole, the manuscript is well-modified. However, there are two small flaws that may need attention. First, L156-146, If patients reported pain ≥ 4 on the numerical rating scale (NRS), 5-ug boluses of sufentanil were given. As sufentanil were given, how much time is needed for patient observation? Or, how do you ensure that the extra sufentanil does not affect the dosage of the PCIA?

**Response:**

We thank the reviewer for the comments regarding postoperative analgesia, which should be described in the manuscript. If patients reported pain ≥ 4 on the numerical rating scale (NRS), 5-ug boluses of sufentanil were given in PACU. After 15 min,  patients’ pain intensity would be assessed again. When Intensity of pain by NRS was less than 4 points and the vital signs was stable, the patient would be transferred to the surgical ward. In fact, because of intraoperative opioid use, it is rare to present pain NRS ≥4 in the PACU, and in this study there was no colorectal cancer (CRC) patient who reported pain ≥4 in PACU. Therefore, in this study PCIA dosage would not be affected by such analgesia protocol. We have revised the related descriptions in the revision manuscript accordingly. (Page7, line147-150; Page12, line245-247)

2. Second, though there was no significant difference with respect to the incidence of nausea and vomiting, but no antemetic were used in PCIA in both the two groups. Is this inappropriate for the patient recovery?

**Response:** We thank the reviewer for the comments regarding postoperative analgesia, which should be described in the manuscript. Actually, in our hospital all patients would be routinely injected intravenously metoclopramide 10 mg and dexamethasone 5 mg before anesthesia induction, in order to prevent intraoperative stress response and postoperative nausea. We have added this related descriptions in the revised manuscript. (Page7, line136-138)

**Reviewer #3:**

1.The sample size calculation can still not be followed. There are two reasons: a) the statement “According to the set minimum of 0.5 SD” is not clear, in particular without giving the SD. Further the test statistic used as basis for the calculation is not given.

And additionally the total sample size of 120 does not account for a 20% dropout, when 100 patients are intended. This last statement should be given as a calculation error in the limitation section.

**Response:** Thanks for the reviewer for the thoughtful comments and suggestions. We are sorry for the unclear descriptions regarding the sample size calculation. Based on the pilot investigation (n=10), the mean BDI scores at 2 days after CRC surgery was 10.2 (standard deviation [SD] 4.2). The minimum clinically important difference between the experimental and control groups was set as 0.5SD, e.g., 2.1. And the test statistic was based on two sample T-Test (difference). Here because the SD in experimental group is unknown, we assumed it as 3.0. Thus the sample size was calculated based on 10.2±4.2 in control group and assumed 8.1±3.0 in experimental group. With significance set at 0.05 and power set at 80%, the sample size required to detect the assumed differences was about 50 patients, calculated by using PASS 11.0 software (NCSS, LLC. Kaysville, Utah, USA). (Page6, line206-213)

Regarding the sample size calculation, we are sorry for the error, and we have added it in the limitation section. The calculated sample size is estimated to be 20% of the missed follow-up patients, and the total sample size should be 125 but not 120, however no patient was missed in the study and through power calculation based on the primary outcomes (9.9±3.5 *vs.* 7.3±3.4, n=60) the power reached 0.984. (Page22, line392-396)

2. Second the name of the randomization procedure used is still not given.

**Response:** We take the form of simple randomization procedure. (Page6, line115-116)
